# Adherence to the Mediterranean Lifestyle and Desired Body Weight Loss in a Mediterranean Adult Population with Overweight: A PREDIMED-Plus Study

**DOI:** 10.3390/nu12072114

**Published:** 2020-07-16

**Authors:** Cristina Bouzas, Maria del Mar Bibiloni, Alicia Julibert, Miguel Ruiz-Canela, Jordi Salas-Salvadó, Dolores Corella, Maria Dolors Zomeño, Dora Romaguera, Jesús Vioque, Ángel M. Alonso-Gómez, Julia Wärnberg, J. Alfredo Martínez, Luís Serra-Majem, Ramon Estruch, Francisco J. Tinahones, José Lapetra, Xavier Pintó, Antonio García Ríos, Aurora Bueno-Cavanillas, José J. Gaforio, Pilar Matía-Martín, Lidia Daimiel, Vicente Martín-Sánchez, Josep Vidal, Clotilde Vázquez, Emilio Ros, Cesar Ignacio Fernandez-Lázaro, Nerea Becerra-Tomás, Ignacio Manuel Gimenez-Alba, Julia Muñoz, Marga Morey, Alejandro Oncina-Canovas, Lucas Tojal-Sierra, Jéssica Pérez-López, Itziar Abete, Tamara Casañas-Quintana, Sara Castro-Barquero, M. Rosa Bernal-López, José Manuel Santos-Lozano, Ana Galera, Escarlata Angullo-Martinez, F. Javier Basterra-Gortari, Josep Basora, Carmen Saiz, Olga Castañer, Marian Martín, Leyre Notario-Barandiarán, María C. Belló-Mora, Carmen Sayón-Orea, Jesús García-Gavilán, Albert Goday, Josep A. Tur

**Affiliations:** 1 CIBER Fisiopatología de la Obesidad y Nutrición (CIBEROBN), Instituto de Salud Carlos III (ISCIII), 28029 Madrid, Spain; cristinabouvel@gmail.com (C.B.); mar.bibiloni@uib.es (M.d.M.B.); alicia.julibert@uib.es (A.J.); mcanela@unav.es (M.R.-C.); jordi.salas@urv.cat (J.S.-S.); dolores.corella@uv.es (D.C.); mzomeno@imim.es (M.D.Z.); mariaadoracion.romaguera@ssib.es (D.R.); angelmago13@gmail.com (Á.M.A.-G.); jwarnberg@uma.es (J.W.); jalfredo.martinez@imdea.org (J.A.M.); lluis.serra@ulpgc.es (L.S.-M.); restruch@clinic.cat (R.E.); fjtinahones@hotmail.com (F.J.T.); joselapetra543@gmail.com (J.L.); xpinto@bellvitgehospital.cat (X.P.); angarios2004@yahoo.es (A.G.R.); cvazquezma@gmail.com (C.V.); eros@clinic.cat (E.R.); cflazaro@unav.es (C.I.F.-L.); nerea.becerra@urv.cat (N.B.-T.); i.gimenez.alba@valencia.edu (I.M.G.-A.); juliamm02@gmail.com (J.M.); marga.morey@yahoo.es (M.M.); jessicaperezlopez@uma.es (J.P.-L.); iabetego@unav.es (I.A.); tamara.nutricion@gmail.com (T.C.-Q.); scastro@clinic.cat (S.C.-B.); robelopajiju@yahoo.es (M.R.B.-L.); josemanuel.santos.lozano@gmail.com (J.M.S.-L.); agalera@idibell.cat (A.G.); eangullo@ibsalut.caib.es (E.A.-M.); javierbasterra@hotmail.com (F.J.B.-G.); jbasora.tgn.ics@gencat.cat (J.B.); carmen.saiz@uv.es (C.S.); ocastaner@imim.es (O.C.); marian.martin@gmail.com (M.M.); marujabello@gmail.com (M.C.B.-M.); csayon@unav.es (C.S.-O.); jesusfrancisco.garcia@urv.cat (J.G.-G.); Agoday@parcdesalutmar.cat (A.G.); 2 Research Group on Community Nutrition & Oxidative Stress, University of Balearic Islands, Guillem Colom Bldg, Campus, 07122 Palma de Mallorca, Spain; lutojal@hotmail.com; 3 Health Research Institute of the Balearic Islands (IdISBa), 07120 Palma de Mallorca, Spain; 4 Department of Preventive Medicine and Public Health, IdISNA, University of Navarra, 31008 Pamplona, Spain; 5 Universitat Rovira i Virgili, Department of Biochemistry and Biotechnology, Human Nutrition Unit, 43201 Reus, Spain; 6 Institut d’Investigació Sanitària Pere Virgili (IISPV), 43201 Reus, Spain; 7 Department of Preventive Medicine, University of Valencia, 46100 Valencia, Spain; 8 Unit of Cardiovascular Risk and Nutrition, Institut Hospital del Mar de Investigaciones Médicas Municipal d’Investigació Mèdica (IMIM), 08003 Barcelona, Spain; 9 Blanquerna School of Health Sciences, Universitat Ramon Llull, 08022 Barcelona, Spain; 10 Unit of Nutritional Epidemiology, Miguel Hernández University, ISABIAL-UMH, 46020 Alicante, Spain; vioque@umh.es (J.V.); aoncina@umh.es (A.O.-C.); lnotario@umh.es (L.N.-B.); 11 CIBER Epidemiología y Salud Pública (CIBERESP), Instituto de Salud Carlos III (ISCIII), 28029 Madrid, Spain; abueno@ugr.es (A.B.-C.); jgaforio@ujaen.es (J.J.G.); 12 Bioaraba Health Research Institute; Osakidetza Basque Health Service, Araba University Hospital, University of the Basque Country UPV/EHU, 48013 Vitoria-Gasteiz, Spain; 13 Department of Nursing, School of Health Sciences, University of Málaga-IBIMA, 29071 Málaga, Spain; 14 Precision Nutrition Program, IMDEA Food, CEI UAM + CSIC, 28049 Madrid, Spain; 15 Department of Nutrition, Food Sciences, and Physiology, Center for Nutrition Research, University of Navarra, 31008 Pamplona, Spain; 16 Institute for Biomedical Research, University of Las Palmas de Gran Canaria, 35016 Las Palmas de Gran Canaria, Spain; 17 Department of Internal Medicine, IDIBAPS, Hospital Clinic, University of Barcelona, 08036 Barcelona, Spain; 18 Virgen de la Victoria Hospital, Department of Endocrinology, Biomedical Research Institute of Málaga (IBIMA), University of Málaga, 29010 Málaga, Spain; 19 Department of Family Medicine, Research Unit, Distrito Sanitario Atención Primaria Sevilla, 41013 Sevilla, Spain; 20 Lipids and Vascular Risk Unit, Internal Medicine, Hospital Universitario de Bellvitge, Hospitalet de Llobregat, 08907 Barcelona, Spain; 21 Lipids and Atherosclerosis Unit, Department of Internal Medicine, Maimonides Biomedical Research Institute of Cordoba (IMIBIC), Reina Sofia University Hospital, University of Cordoba, 14004 Cordoba, Spain; 22 Department of Preventive Medicine, University of Granada, 18071 Granada, Spain; 23 Department of Health Sciences, Centro de Estudios Avanzados en Olivar y Aceites de Oliva, University of Jaen, 23071 Jaen, Spain; 24 Department of Endocrinology and Nutrition, Instituto de Investigación Sanitaria Hospital Clínico San Carlos (IdISSC), 28040 Madrid, Spain; pilar.matia@gmail.com; 25 Nutritional Genomics and Epigenomics Group, IMDEA Food, CEI UAM + CSIC, 28049 Madrid, Spain; lidia.daimiel@imdea.org; 26 CIBER Diabetes y Enfermedades Metabólicas (CIBERDEM), Instituto de Salud Carlos III (ISCIII), 28029 Madrid, Spain; vicente.martin@unileon.es; 27 Institute of Biomedicine (IBIOMED), University of León, 24071 León, Spain; 28 Department of Endocrinology, IDIBAPS, Hospital Clinic, University of Barcelona, 08036 Barcelona, Spain; jovidal@clinic.cat; 29 Department of Endocrinology, Fundación Jiménez-Díaz, 28040 Madrid, Spain; 30 Lipid Clinic, Department of Endocrinology and Nutrition, Institut d’Investigacions Biomèdiques August Pi Sunyer (IDIBAPS), Hospital Clínic, 08036 Barcelona, Spain; 31 Escola Graduada Primary Health Care Center, IBSalut, 07002 Palma de Mallorca, Spain; 32 Servicio Navarro de Salud, Osasunbidea, 31071 Pamplona, Spain

**Keywords:** body image, mediterranean lifestyle, overweight, obesity, older adults, desired weight loss, ideal weight, PREDIMED-Plus

## Abstract

Background. Body weight dissatisfaction is a hindrance to following a healthy lifestyle and it has been associated with weight concerns. Objectives. The aim of this study was to assess the association between the adherence to the Mediterranean lifestyle (diet and exercise) and the desired body weight loss in an adult Mediterranean population with overweight. Methods. Cross-sectional analysis in 6355 participants (3268 men; 3087 women) with metabolic syndrome and BMI (Body mass index) between 27.0 and 40.0 kg/m^2^ (55–75 years old) from the PREDIMED-Plus trial. Desired weight loss was the percentage of weight that participants wished to lose. It was categorized into four cut-offs of this percentage (Q1: <10%, *n* = 1495; Q2: 10–15%, *n* = 1804; Q3: <15–20%, *n* = 1470; Q4: ≥20%, *n* = 1589). Diet was assessed using a validated food frequency questionnaire and a 17-item Mediterranean diet questionnaire. Physical activity was assessed by the validated Minnesota-REGICOR and the validated Spanish version of the Nurses’ Health Study questionnaire. Results. Participants reporting higher percentages of desired weight loss (Q3 and Q4) were younger, had higher real and perceived BMI and were more likely to have abdominal obesity. Desired weight loss correlated inversely to physical activity (Q1: 2106 MET min/week; Q4: 1585 MET min/week. *p* < 0.001) and adherence to Mediterranean diet (Q1: 8.7; Q4: 8.3. *p* < 0.001). Conclusions. In older Mediterranean individuals with weight excess, desired weight loss was inversely associated with Mediterranean lifestyle adherence. Deeply rooted aspects of the MedDiet remained similar across groups. Longitudinal research is advised to be able to establish causality.

## 1. Introduction

Despite a recent meta-analysis describing that overweight or obesity may decrease mortality risk in elderly populations [1], this could be due to a higher resistance of the overweight people to severe physical stress, as would be the case for inpatients in intensive care units [2]. On the other hand, sarcopenic obesity increased risk for all-cause mortality [3]. Ju et al. described that other parameters closely related to obesity, such as metabolic syndrome, increase mortality. However, they do not describe in detail parameters of body composition when they refer to weight excess as a protective factor for mortality [1]. Moreover, the excess of body weight and body fat is associated with a higher risk of several diseases, which are direct causes of a decrease in quality of life and mortality [4,5,6,7,8]. Hence, body fat should be diminished to adequate levels, in order to avoid such outcomes. Aging comes to relevance especially since prevalence of non-communicable chronic diseases, such as those related to an excess of weight or susceptible of aggravating by weight, increases after 55 years of age [9]. Previous research established that the Mediterranean diet (MedDiet) is an adequate treatment to avoid cardiovascular harmful effects of excess weight and its comorbidities in population over 55 years old [10]. Several definitions of MedDiet are available through the literature. Reviews in this regard have found similarities and differences among definitions. Briefly, the MedDiet is a food pattern rich in fruits, vegetables, olive oil, whole grains, legumes, nuts, fish and a preference of white over read meat. Less consistent are the recommendations for fermentable dairy products and red wine in the literature [10,11,12,13,14,15,16]. From a holistic point of view, the MedDiet has been considered one of the key elements of the so-called Mediterranean lifestyle, which moreover adds to the diet qualities of eco-friendly and sustainability by the preference for locally produced, traditional and seasonal foods. Further from food itself, it also implies correct hydration, home-made food preparation, sociable eating with family or friends, regular outdoor physical activity, relaxation and rest [14,15].

Rather than the objective weight status, weight perception and ideal weight are more likely to boost weight management actions [16], as illustrated by Higgins’ regulatory focus theory regarding the relationship between motivations and pursuit of a goal [17]. Accordingly, overweight perception has been associated with spontaneous weight management, mainly by dieting and/or exercising [16]. Nowadays, checking for health advice in social media has become very popular [18]. Searches comprise a wide range, from searching symptoms online with the purpose of self-diagnosis, to self-prescribing a treatment [19]. There is an increasing tendency to self-prescribe a diet [19], however, unfortunately not all information available on internet is reliable [18]. As a consequence, body weight dissatisfaction is a risk factor for engaging in unhealthy lifestyles, and it might be a hindrance to following a healthy lifestyle [16]. Nonetheless, aging has been associated with lower weight concerns and lower overweight perception. Ignoring and not tackling the excess of fat may have a negative impact on lifestyle and general health, as mentioned above [4,16]. Moreover, aging has been associated with lower discrepancy between current weight and desired weight [20]. Thus, it is unclear whether aging offers a protective or harmful effect on the influence of ideal weight on healthful lifestyles. The lower concern might protect individuals from unreliable health information, while a decrease in awareness might decrease interest in maintaining a healthy lifestyle.

Body image (defined as a person’s perception of their physical self [21]) has been widely studied in the young population, but it has been scarcely reported on in the aged population [16]. Body image can be assessed as body image dissatisfaction, by comparing actual and reported desired body weight [22]. On one hand, reported weight and desired weight are shifting upward, contrary to the percentage of desired weight loss (DWL), which remained more stable through time. [23,24]. As aforementioned, evidence tackling the relationship between desired weight or DWL and dietary pattern in middle aged and aged populations without eating disorders is very limited. It would be interesting to study such associations in adults with overweight. The PREDIMED-Plus study offers a golden opportunity to evaluate relations between body image defined as DWL and Mediterranean lifestyle in adults over 55 years old. Therefore, the aim of this study was to assess the association between the adherence to Mediterranean lifestyle (understood as diet and exercise) and the desired body weight loss in an adult Mediterranean population with an excess of weight.

## 2. Methods

### 2.1. Study Design

The PREDIMED-Plus trial is an ongoing 6-year multicenter, parallel-group, randomized trial. It is currently being conducted in 23 Spanish recruiting centers (universities, hospitals and research institutes). The PREDIMED-Plus trial was designed to compare the effect of a hypocaloric traditional MedDiet combined with physical activity promotion and behavioral support on cardiovascular disease morbimortality, compared with the usual care advice, consisting exclusively of an energy-unrestricted traditional MedDiet (control group). Further details on the study protocol can be found elsewhere [25] and at http://predimedplus.com/. The trial was registered in 2014 at the International Standard Randomized Controlled Trial (ISRCT; http://www.isrctn.com/ISRCTN89898870) with number 89898870. This present research is a cross-sectional analysis of baseline data within the frame of the PREDIMED-Plus trial. Because the present research is a cross-sectional analysis of baseline data, no differences were made in the analysis by treatment group allocation.

### 2.2. Participants, Recruitment and Ethics

**Figure 1:**
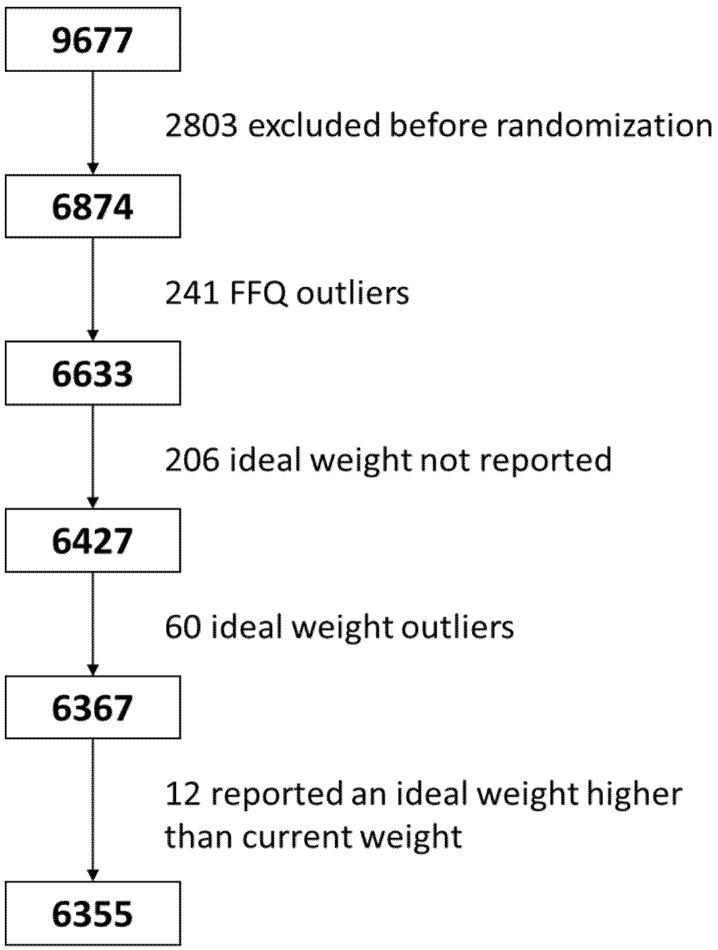
Flow chart of participants.

A total of 9677 people were contacted, of which 6874 participants were eligible for the study, and were included in the trial (Figure 1). Eligible participants were community-dwelling adults (men aged 55–75, women aged 60–75), who were overweight or obese (body mass index (BMI) between 27.0 and 40.0 kg/m^2^) and meeting at least three criteria for metabolic syndrome according to the updated harmonized definition of the International Diabetes Federation, the American Heart Association and the National Heart, Lung and Blood Institute [26]. All participants provided written informed consent, and the study protocol and procedures were approved according to the ethical standards of the Declaration of Helsinki by all the participating institutions.

### 2.3. Dietary Assessment

Registered dietitians assessed baseline dietary habits through dietary intake obtained with a semi quantitative 143-item food frequency questionnaire (FFQ) [27] which has been previously validated in the Spanish population [27,28,29]. For each item, a regular portion size was established, and consumption frequencies were registered in 9 categories, ranging from “never or almost never” to “≥6 times/day”. Energy and nutrient intakes were calculated as frequency multiplied by nutrient composition of specified portion size for each food item, using a computer program based on available information in Spanish food composition tables [30,31]. Intake of dietary supplements declared in the FFQ was also considered when assessing the total nutrient intake. Participants reporting extreme total energy intakes (<500 or >3500 kcal/day in women or <800 or >4000 kcal/day in men) were excluded from the analysis [32]. Because 241 participants reported extreme total energy intakes; therefore, our study sample was reduced to 6633 subjects.

### 2.4. MedDiet Adherence Assessment

Adherence to Mediterranean dietary patterns was assessed by a modified version of the previously validated questionnaire used in the PREDIMED trial. Registered dietitians administered the 17-item MedDiet (17-item erMedDiet) questionnaire measuring adherence to an energy-restricted MedDiet [33,34] in which each item is related to a food habit (see Table 1). Compliance with food habits scored 1 for every item, otherwise scored 0. Therefore, the 17-item MedDiet questionnaire ranged between 0 and 17. Tertiles were made to define low, moderate or high adherence, ranging from 0 to 7, 8 to 10, and 11 to 17, respectively, as previously published [34].

### 2.5. Desired Weight-Loss (DWL)

An eating disorder questionnaire [25] was administered at baseline. The questionnaire aimed to detect comorbid eating disorders according to DSM-IV criteria [35]. On it perceived weight and height, as well as maximum and minimum weight, were asked. Moreover, reported ideal weight (expressed in Kg) was asked to the participants in the aforementioned questionnaire. Weight and height were measured in duplicate by registered dietitians with calibrated scales (BC 418 MA Body Composition Analyzer/Scale, Tanita, Tokyo, Japan) and a wall-mounted stadiometer (Seca 213, HealthCheck Systems, Brooklyn, NY, USA), respectively. BMI was calculated as weight in kilograms divided by the square of height in meters. A total of 206 participants who did not report a subjective ideal weight were excluded from the analysis; therefore, the sample was reduced to 6427. Actual BMI was obtained with measured weight and height, while perceived BMI was calculated with reported (perceived) weight and height.

The literature has described that desired weight and reported weight are shifting upward. Nevertheless, when desired weight was examined as the percentage of body weight, such tendencies were not found [23,24]. Therefore a new variable was computed by subtracting subjective ideal weight from measured weight at baseline. Outliers (defined as 3 or more standard deviations (SD) from both sides of the mean) of that variable were excluded from the analysis. 60 outliers were found, therefore the sample size reduced from 6427 to 6367. If the former variable ranged between 2 and −2 kg, authors considered that subjective ideal and objective measured weight were similar [36,37]. Only 12 subjects reported higher desired than current weight. Since all participants were obese or overweight (BMI between 27.0 and 40.0), those twelve subjects were also excluded from the analysis. Therefore, the final sample included 6355 subjects, 3268 men and 3087 women.

The desired weight-loss at baseline (DWL) was the weight that each participant would need to lose to reach their subjective ideal weight. In the present study, DWL was expressed as a percentage (percentage of weight that they wish to lose). DWL was obtained through the following equation:

(1) $$\mathrm{DWL}\left(\%\;\mathrm{desired}\;\mathrm{weight}\;\mathrm{loss}\right)=\frac{\left(\mathrm{baseline}\;\mathrm{weight}\;-\;\mathrm{ideal}\;\mathrm{weight}\right)}{\mathrm{baseline}\;\mathrm{weight}}\times 100$$

Subjects were initially categorized into quartiles of the absolute value of DWL for analysis. Due to the closeness of the cutting-percentiles (p75: 20.00%; p50: 14.65%; p25: 10.25%), cut-offs were made considering a 5% increase in DWL (Q1: <10% of DWL, *n* = 1495; Q2: 10–15% of DWL, *n* = 1804; Q3: <15–20% of DWL, *n* = 1470; Q4: ≥20% of DWL, *n* = 1589), which would make it easier to transfer results to everyday clinical practice.

### 2.6. Other Variables

Information related to smoking habits, marital status, educational level, as well as medical history and current medication were obtained. Biochemical analyses (triglycerides, total cholesterol, HDL-cholesterol and fasting plasma glucose) were performed using overnight fasting blood samples by standard enzymatic methods. Blood pressure was measured in triplicate with a validated semi-automatic oscillometer (Omron HEM, 705CP, Hoofddrop, The Netherlands) in a seated position. Waist circumference was measured in duplicate, halfway between the last rib and the iliac crest by using an anthropometric tape.

The validated Minnesota-REGICOR short physical activity questionnaire [38,39,40] and the validated Spanish version of the Nurses’ Health Study questionnaire [41] were used to assess physical activity and sedentary behaviors, respectively.

### 2.7. Statistics

Analyses were performed with the SPSS statistical software package version 25.0 (SPSSS Inc., Chicago, IL, USA). Data are shown as mean, standard deviation (SD) and median, interquartile range (IQR). Differences among groups were tested with one-way ANOVA and Bonferroni’s post-hoc analysis when variables followed normal distribution, or Kruskal–Wallis models in other cases. Prevalence is expressed in sample size and percentage. Difference in prevalence among groups was tested using χ^2^ (all *p* values are two-tailed). Multivariate analysis was used to assess association between the MedDiet 17 items (dependent variables) and percentage (cut-off) of desired body weight loss (independent variables). For each item, 3 Odds Ratio (OR) were calculated: crude, adjusted by sociodemographic factors (age, BMI, physical activity, diet, education level, marital status and smoking habit), and adjusted by both sociodemographic factors and presence of metabolic syndrome components.

## 3. Results

Table 2 shows sociodemographic characteristics according to cut-offs of DWL. Participants with higher DWL (Q3 and Q4) were younger, had higher BMI (actual and perceived) and higher rates of abdominal obesity. No other components of the metabolic syndrome were different among groups except for high blood pressure and hyperglycemia in women (Supplemental Tables S1 and S2). A total of 27% of the subjects were overweight while 73% were obese. The majority of the subjects with overweight were classified into Q1 (52%) and Q2 (35%). On the contrary, most of the subjects with obesity were in Q4 (33%), Q3 (28%) and Q2 (27%). Moreover, 68%, 87% and 97% of the subjects in quartile 2, 3 and 4, respectively, had obesity. Tackling lifestyle, Q4 registered the lowest physical activity rates (Q1: 2106 MET min/week; Q4: 1585 MET min/week. *p* < 0.001). Although there was no difference in total energy intake according to DWL, adherence to the MedDiet decreased as DWL increased (Q1: 8.7; Q4: 8.3. *p* < 0.001). Q1 had fewer participants living alone and more married participants than the other groups. This was especially significant among women. In women, higher DWL was related to higher education levels, as well as to higher likelihood of ever smoking, but also to higher rates of abandoning tobacco consumption (Supplemental Tables S1 and S2).

MedDiet adherence evaluated with the 17-item MedDiet questionnaire is available in Table 3. Low MedDiet adherence scores were more likely to be found among participants with higher DWL (Q1: 32.6%; Q4: 37.8%. *p* = 0.007), as opposed to high scores, more easily found as DWL decreased (Q1: 25.7%; Q4: 20.6%. *p* = 0.007). Vegetables, fruits, nuts, red and processed meat, and sugary sweetened beverages were the most relevant items decreasing overall adherence to the MedDiet. Conversely, avoiding adding sugar to beverages was higher among Q4. Tackling genders (Supplemental Tables S3 and S4), items decreasing adherence for men were those regarding vegetables, fish and seafood and preference of white over red meat; while for women were those regarding fruits, red and processed meat, adding sugar to beverages or consumption of sugary sweetened beverages, and using olive oil for cooking.

Lastly, crude and adjusted OR for adherence to the 17 item MedDiet questionnaire items across cut-off Q1–4 of DWL are presented in Table 4. Q1 (<10% DWL) was established as the reference. Crude and adjusted analysis shows that OR for Q3 and Q4 was 0.75–0.85 times lower than Q1 for adhering to the items regarding vegetables, fruits, red and processed meat, and sugary sweetened beverages; and 0.65–0.80 times lower for nuts. On the other hand, avoiding adding sugar to beverages in Q4 had a crude OR 1.25 times higher than the Q1, but it disappeared after adjustment. Some associations (crude OR) were found only for one gender (Supplemental Tables S5 and S6). In men, Q4 had an OR 0.75–0.80 times lower than Q1 to meet the recommendations of fish or seafood and to prefer white over red meat. In women, Q4 had an OR 1.35–1.40 times higher than Q1 to use extra virgin olive oil for cooking.

Some associations were modified due to adjustment by potential confounders. While OR for items regarding vegetables, red meat and nuts remained similar to crude OR (between 0.6–0.9 times lower for Q4 than Q1), fruits and sugary sweetened beverage items lost their statistical significance due to adjustment. OR for consuming white bread was 0.80–0.85 times lower for Q2 than for Q1, and, only in women also for Q4. On the other hand, men in Q4 had an OR 0.68 times lower for consuming less than 3 portions of refined cereals per week than those in Q1. Regarding the use of extra virgin olive oil for cooking, Q2 and Q4 had an OR 1.3 times higher, while for women alone OR for Q4 increased up to 1.6. The OR of drinking wine changed after adjustment only by sociodemographic factors, Q2 (10–15% desired weight loss) has an OR of 1.27.

## 4. Discussion

In the present study, DWL was inversely associated with Mediterranean lifestyle (diet and physical activity) and directly associated with BMI and abdominal obesity. Previous studies reported that BMI was associated with a higher discrepancy between current body weight and subjective ideal weight [24]. Regardless of the exception of African American women, ideal weight tends to fall within the normal weight range [42,43]. This supports our hypothesis that higher DWL in people with overweight, especially when some comorbidities are present, might be motivated by a high current weight and a desire to improve health. Moreover, a recent study showed that in normal weight individuals, overestimation of weight status together with diagnosis of metabolic syndrome, increases weight loss efforts [44]. This health motivator might also be an explanation of the finding that, in women, percentage of former smokers increased as DWL increased.

In the present study, higher DWL were related to lower levels of physical activity, which was consistent with existing literature [24,45,46,47,48,49,50]. High weight perception has been associated with lower levels of physical activity in adults [51], however current results are controversial and no definite conclusion can be drawn in this regard [50]. Some hypothesis have been made to explain these relationships, such as negative evaluations to develop exercise in public [52,53], or that physical activity modified body perception and helped to maintain a satisfactory body image [54,55], and therefore lower DWL. Self-perception has been described as a motivator for senior women to start exercising [56]; nonetheless, exercise would help weight management and therefore decrease DWL.

Our findings show no relationships between energy intake and DWL, unlike existing literature, which has associated body image dissatisfaction to high energy intake [45]. A plausible explanation would point out that DWL might affect energy intake reporting; however, there is little and yet mixed evidence on the relation between energy intake underreporting and ideal weight. While for adults lower ideal than current weight has been associated with underreporting 339 kilocalories per day [57], in women aged 50–75 years old there were no associations with underreporting energy [58]. Therefore, it cannot be assumed that DWL is affecting energy intake reporting.

The available literature on the topic is consistent with our results regarding food consumption and DWL. On one hand, having a large body image has been associated with unhealthier dietary patterns, such as higher intakes of sweet drinks and refined foods. On the other hand, small body image has been associated to a healthy dietary pattern, rich in fruits and vegetables [59]. Body dissatisfaction caused by an excess of weight has also been associated with unhealthy eating habits, such as ultra-processed foods [60]. In this regard, the present study found that as DWL increased, so did sugar sweetened beverage consumption, especially in women, as well as avoidance to add sugar to beverages also increased, mainly in men. Considering DWL as a source of stress, chocolates, biscuits, cakes, sweets and palatable snacks were consumed more frequently under stress, as opposed to fish, meat, fruits and vegetables, which are the less consumed foods under stress [61]. Furthermore, low ideal BMI has been associated with weight management [16]. Lowering fat consumption is a technique that men are likely to apply to lose weight [62]. This could explain why nuts consumption was lower among those in the Q4. Moreover weight management has been associated with a higher consumption of fruits and vegetables, especially in women [24,63,64]. The low consumption of fruits and vegetables in Q4 could be related to low pursuit of their ideal weight. This theory is supported by existing literature, stating that compared with population aged under 40 years old, those over 60 settled for lower weight loss expectations [65] and therefore had a lower pursuit of weight control.

Although some research was unsure about the adequacy of the MedDiet as a long term weight loss method [66], more recent evidence has shown that the MedDiet is a valid strategy for long term weight management [67,68,69], and is, moreover, effective in reducing obesity adverse health consequences [3]. In the present study, DWL was inversely associated to Mediterranean lifestyle adherence. Those findings align with the existing literature on the topic, which has associated weight dissatisfaction with less healthy lifestyles, understood as healthy diet and exercise, contrary to weight satisfaction that related to healthier lifestyles [24,45,70]. Weight dissatisfaction was also related to greater intention to change lifestyle [24]. Moreover, identifying oneself as part of a socially stigmatized group also may promote less healthy dietary habits [70,71] On the other hand, as suggested above, if bigger DWL in this population were primarily motivated by health pursuits, they might not be capable of spontaneously following a healthy lifestyle, which would improve general health.

The present population is living in a Mediterranean country, immersed in the Mediterranean culture and lifestyle; but at the same time, the current globalization is spreading the influence of the western eating style [72]. The MedDiet has been described as a dietary pattern characterized by a high intake of olive oil as the main source of culinary fat, and high intakes of vegetables, fruits, nuts, legumes and fish, at the expense of a lower intake of meat [13], while the western-style diet is a high-calorie pattern rich in refined wheat, meat, and sodas and a low intake of legumes, nuts, fish, fruits and vegetables [11,72]. Bearing in mind the present results regarding compliance with MedDiet items and DWL, it could be suggested that DWL might have a higher impact on those dietary items or food groups for which the Western and Mediterranean patterns differ the most, such as fruits, vegetables, nuts, red meat and sodas. On the other hand, those aspects of the MedDiet assumed to be more cultural, such as “sofrito” making, legumes, or fish consumption were the items that remained more stable among groups of DWL, or, as happened for olive oil, even improved adherence in some groups, also supporting the health pursuit theory. Therefore, the dietary patterns of participants reporting higher DWL might be more influenced by the western eating style, altogether with lower dietary quality and physical activity, than subjects with lower DWL.

The group with higher DWL was the youngest. Aging has been related to lower weight loss expectations [65] and to healthier diet [73,74]. In our study, analysis was adjusted by age, hence, we can assume that the associations found between diet and DWL are not affected by age.

### Strengths and Limitations of the Study

The present study contributes to the very limited evidence tackling the relationship between dietary lifestyle and body image in populations aged over 55 years old. Other strengths of the present study include its large sample size and the use of two different tools to assess dietary intake: the FFQ and the 17-item MedDiet. On the top of that, results would be very easily transferred into clinical practice, as groups were defined within 5% of desired weight loss. This classification makes it easier to transfer results to everyday clinical practice. 

Nonetheless, the present study has some limitations. The main limitation would be that causal inferences cannot be established, as it has a cross-sectional design. Secondly, it has been described that, when starting a weight loss program, ideal weight is lower than real weight and related to maximum weight loss previously achieved [75,76]. Therefore, not taking into consideration realistic weight losses is the second limitation of the present study. In addition, body image is a multidimensional construct that is hard to simplify [77]. The authors are aware that there are other validated methods to assess body image and dissatisfaction [78,79] that were not used in the present research. In the present work we tried to simplify the assessment of body image through surrogate parameters that are easily obtained in clinical practice. This was done to allow transference of present findings to clinical practice. Thirdly, FFQ, even after being validated, might overestimate intake of certain food groups. For all that, participants reporting extreme energy intakes were excluded, and the 17-item MedDiet was used to contrast, to avoid information bias [80]. Moreover, due to the lack of data collection, the influence of economic status could not be evaluated as a confounder. Lastly, all participants in the present study were over 55 years and about to start a healthier lifestyle as part of the PREDIMED-Plus trial, and had high cardiovascular risk, which is a limitation to make results extensible to the general adult population.

## 5. Conclusions

Following Mediterranean lifestyle is beneficial for general health, especially for those who are already at risk due to an excess of weight. The present study showed that in a population with an excess of weight aged over 55 years, DWL inversely correlated to Mediterranean lifestyle, by adherence to MedDiet and levels of physical activity. As DWL increased, food intake shifted to low dietary quality, through an unhealthy dietary pattern rich in processed foods and sugary sweetened beverages, and low intake of fruits and vegetables. The most rooted aspects of the MedDiet remain stable regardless of the DWL. Moreover, physical activity decreased as DWL increased. DWL could be a tool for health care professionals to detect whether a person is at risk due to an unhealthy lifestyle. As it has been related to lower physical activity and specific diet components, those should be specially addressed in further detail by health care professionals. More research is needed in this regard, to validate and further define the potential tool.

The present study increases the little evidence regarding physical self-perception in older adults. Further research on DWL and lifestyle ought to be conducted. To be able to establish causality, longitudinal design is advised. It is necessary to explore if the less healthy lifestyle is influencing DWL or if it is the other way around, to be able to design more effective weight management strategies.

## Figures and Tables

**Table 1:** Description of the 17-item Mediterranean dietary questionnaire.

	Item	Compliance with the Item
*1*	Extra-virgin olive oil for cooking	Use only extra-virgin olive oil for cooking, salad dressings, and spreads.
*2*	Vegetables	Consume ≥2 portions (200 g) of vegetables per day, at least one of them raw.
*3*	Fruits	Consume ≥3 portions of fruit per day (including natural fruit juices).
*4*	Red and processed meat	Consume ≤1 serving (100–150 g) of red meat, hamburgers, or meat products (ham, sausage, etc.) per week.
*5*	Butter, margarine, cream.	Consume less than 1 serving (12 g) of butter or cream per week.
*6*	Sugar sweetened beverages	Consume less than one sugary beverage or sugar-sweetened fruit juice per week.
*7*	Legumes	Consume ≥3 servings (150 g) of legumes per week.
*8*	Fish and seafood	Consume ≥3 servings of fish (100–150 g) or shellfish (200 g) per week.
*9*	Sweets and pastries	Consume <3 non-homemade sweets or pastries, such as cakes, cookies, sponge cake, or custard, per week.
*10*	Nuts	Consume ≥3 servings of nuts (including peanuts) per week.
*11*	Preference white over red meat	Consume chicken, turkey or rabbit meat instead of beef, pork, hamburgers or sausages.
*12*	Sofrito	Use sofrito ≥2 times per week (Sofrito: tomato and onion sauce, with garlic and aromatic herbs, simmered in olive oil.).
*13*	Adding sugar to beverages	Replace sugar with non-caloric artificial sweeteners for beverages.
*14*	White bread	Reduce consumption of white bread to >1 serving (75 g)/day.
*15*	Whole grains	Consume whole grain cereals and whole grain pasta ≥ 5 times per week.
*16*	Refined cereals	Reduce consumption of non-whole grain pasta or rice < 3 servings per week.
*17*	Wine	Consume 2–3 glasses (200 mL/glass) of wine per day (men) or 1–2 glasses of wine per day (women).

**Table 2:** Sociodemographic characteristics according to percentage of desired weight loss (DWL).

	Q1 § (*n* = 1492)		Q2 § (*n* = 1804)		Q3 § (*n* = 1470)		Q4 § (*n* = 1589)		
	Mean (SD)	Median (IQR)	Mean (SD)	Median (IQR)	Mean (SD)	Median (IQR)	Mean (SD)	Median (IQR)	*P*
Age (years)	65.9 (5.0)	66.0 (8.0) ^a,b,c^	65.0 (4.8)	65.0 (8.0) ^a,e^	64.8 (4.9)	65.0 (7.0) ^b,f^	64.3 (4.7)	64.0 (7.0) ^c,e,f^	<0.001
BMI (kg/m^2^)	29.8 (2.2)	29.5 (2.9) ^a,b,c^	31.5 (2.8)	31.2 (3.7) ^a,d,e^	33.2 (2.9)	33.0 (4.0) ^b,d,f^	35.5 (2.9)	35.5 (4.3) ^c,e,f^	<0.001
Perceived BMI (kg/m^2^)	30.0 (2.7)	29.6 (3.1) ^a,b,c^	31.5 (2.9)	31.2 (3.9) ^a,d,e^	33.2 (3.3)	32.8 (4.1) ^b,d,f^	35.4 (3.3)	35.4 (4.6) ^c,e,f^	<0.001
**Physical activity (PA) †**									
Total (PA) †	2730.8 (2459.4)	2106.3 (2540.8) ^b,c^	2583.7 (2242.9)	2055.9 (2641.6) ^d,e^	2387.7 (2311.2)	1762.7 (2454.6) ^b,d,f^	2173.4 (2194.7)	1585.1 (2394.4) ^c,e,f^	<0.001
Light PA †	792.8 (974.7)	447.6 (1118.9)	753.9 (940.5)	447.6 (1118.9)	761.5 (949.6)	447.6 (1118.9)	758.8 (957.2)	447.6 (1118.9)	0.667
Moderate PA †	1089.1 (1615.8)	507.0 (1573.4) ^b,c^	1029.0 (1543.8)	338.0 (1573.4) ^e^	906.2 (1534.2)	279.7 (1398.6) ^b,f^	758.7 (1374.1)	0.0 (1049.0) ^c,e,f^	<0.001
Intense PA †	848.8 (1633.4)	86.7 (1118.9) ^c^	800.8 (1370.7)	111.9 (1118.9) ^d,e^	720.0 (1384.8)	83.9 (925.4) ^d^	655.8 (1277.3)	55.9 (839.2) ^c,e^	<0.001
**Diet**									
Energy intake (kcal/d)	2361.1 (535.8)	2334.4 (708.6)	2368 (541.7)	2326 (733.4)	2376.2 (555.6)	2350.3 (771.2)	2356.4 (569.5)	2318.7 (780.0)	0.711
MedDiet Q score	8.7 (2.7) ^b,c^	9.0 (4.0)	8.5 (2.7)	8.0 (3.0)	8.4 (2.6) ^b^	8.0 (4.0)	8.3 (2.6) ^c^	8.0 (4.0)	<0.001
	*n* (%)		*n* (%)		*n* (%)		*n* (%)		
Gender (female)	662 (21.4)		837 (23.0)		709 (23.0)		879 (28.5)		<0.001
**BMI classification**									<0.001
Overweight (BMI < 30)	877 (51.4)		588 (34.4)		190 (11.1)		52 (3.0)		
Obesity (BMI > 30)	615 (13.4)		1214 (26.4)		1267 (27.6)		1502 (32.7)		
**Education level**									0.089
Primary	773 (52.1)		852 (47.5)		684 (47.1)		778 (49.5)		
Secondary	399 (26.9)		528 (29.4)		448 (30.9)		462 (29.4)		
Tertiary	312 (21.0)		413 (23.0)		320 (22.0)		332 (21.1)		
**Smoking habit**									0.177
Current smoker	174 (11.7)		241 (13.4)		179 (12.3)		195 (12.3)		
Former smoker	617 (41.6)		770 (42.8)		661 (45.2)		712 (44.9)		
Never smoked	693 (46.7)		787 (43.8)		621 (42.5)		678 (42.8)		
**Marital status**									0.001
Married	1172 (79.0)		1391 (77.3)		1123 (76.6)		1174 (74.1)		
Divorced/separated	89 (6.0)		143 (7.9)		116 (7.9)		140 (8.8)		
Widower	173 (11.7)		166 (9.2)		151 (10.3)		175 (11.0)		
Other ‖	50 (3.4)		99 (5.5)		76 (5.2)		96 (6.1)		
Living alone ‡	155 (10.4)		238 (13.2)		176 (12.0)		211 (13.3)		0.047
**Smoking habit**									0.177
Current smoker	174 (11.7)		241 (13.4)		179 (12.3)		195 (12.3)		
Former smoker	617 (41.6)		770 (42.8)		661 (45.2)		712 (44.9)		
Never smoked	693 (46.7)		787 (43.8)		621 (42.5)		678 (42.8)		
**MetS components**									
High blood pressure	1373 (92.0)		1643 (91.1)		1368 (93.1)		1462 (92.0)		0.227
Hyperglycemia	1138 (76.3)		1330 (73.7)		1096 (74.6)		1231 (77.5)		0.056
Hypertriglyceridemia	835 (56.0)		1000 (55.4)		837 (56.9)		869 (54.7)		0.644
Low HDL-cholesterol	657 (44.0)		752 (41.7)		604 (41.1)		694 (43.7)		0.262
Abdominal obesity	1341 (89.9)		1735 (96.2)		1445 (98.3)		1585 (99.7)		<0.001

Abbreviations: BMI. Body Mass Index. PA. Physical activity. MedDiet Q. 17-item Mediterranean Diet Questionnaire HDL-cholesterol. High density lipoprotein cholesterol. § Desired body weight loss = [(current body weight − ideal body weight)/current body weight] × 100. Due to the closeness of the cutting-percentiles, cut-offs were made considering a 5% of increase in desired body weight: Q1: <10% desired body weight loss; Q2: 10–15% desired body weight loss; Q3: 15–20% desired body weight loss; Q4: ≥20% desired body weight loss. † Measured in MET (Metabolic equivalent of task) min/week. ‖ Other marital status were single and religious. ‡ Living alone regardless of marital status. Difference in means between groups were tested by one-way ANOVA and Bonferroni’s post-hoc when normally distributed or Kruskal-Wallis test when otherwise. Differences in prevalence’s across groups were examined using χ^2^. Different letters indicate statistically significant differences between groups (a–f) according to Bonferroni’s post-hoc analysis.

**Table 3:** Adherence to the Mediterranean Diet 17-items according to the percentage of desired weight loss (DWL).

	Q1 § (*n* = 1492)	Q2 § (*n* = 1804)	Q3 § (*n* = 1470)	Q4 § (*n* = 1589)	
**MedDiet 17-items**	*n* (%)	*n* (%)	*n* (%)	*n* (%)	*P*
1: EVOO for cooking	1152 (77.2)	1454 (80.6)	1151 (78.3)	1262 (79.4)	0.100
2: Vegetables	609 (40.8)	631 (35.0)	526 (35.8)	546 (34.4)	0.001
3: Fruits	758 (50.8)	829 (46.0)	675 (45.9)	698 (43.9)	0.001
4: Red and processed meat	772 (51.7)	856 (47.5)	654 (44.5)	745 (46.9)	0.001
5: Butter, margarine, cream.	1209 (81.0)	1460 (80.9)	1161 (79.0)	1246 (78.4)	0.149
6: Sugar sweetened beverages	1155 (77.4)	1354 (75.1)	1081 (73.5)	1162 (73.1)	0.029
7: Legumes	297 (19.9)	341 (18.9)	262 (17.8)	306 (19.3)	0.532
8: Fish and seafood	690 (46.2)	875 (48.5)	684 (46.5)	714 (44.9)	0.215
9: Sweets and pastries	908 (60.9)	1079 (59.8)	871 (59.3)	905 (57.0)	0.151
10: Nuts	650 (43.6)	814 (45.1)	550 (37.4)	545 (34.3)	<0.001
11: Preference white over red meat	1114 (74.7)	1325 (73.4)	1092 (74.3)	1177 (74.1)	0.879
12: Sofrito	855 (57.3)	1036 (57.4)	826 (56.2)	885 (55.7)	0.701
13: Adding sugar to beverages	940 (63.0)	1104 (61.2)	945 (64.3)	1080 (68.0)	0.001
14: White bread	688 (46.1)	776 (43.0)	679 (46.2)	725 (45.6)	0.198
15: Whole grains	418 (28.0)	481 (26.7)	416 (28.3)	412 (25.9)	0.398
16: Refined cereals	486 (32.6)	534 (29.6)	449 (30.5)	473 (29.8)	0.250
17: Wine	342 (22.9)	463 (25.7)	329 (22.4)	317 (19.9)	0.001
**MedDiet Adherence**					0.007
Low adherence (0–7)	487 (32.6)	655 (36.3)	550 (37.4)	600 (37.8)	
Moderate adherence (8–10)	621 (41.6)	719 (39.9)	591 (40.2)	662 (41.7)	
High adherence (11–17)	384 (25.7)	430 (23.8)	329 (22.4)	327 (20.6)	

§ Desired body weight loss = [(current body weight − ideal body weight)/current body weight] × 100. Due to the closeness of the cutting-percentiles, cut-offs were made considering a 5% of increase in desired body weight: Q1: <10% desired body weight loss; Q2: 10–15% desired body weight loss; Q3: 15–20% desired body weight loss; Q4: ≥20% desired body weight loss. Differences in prevalence’s across groups were examined using χ^2^.

**Table 4:** Association between the adherence to the Mediterranean Diet 17-items (dependent variables) and the percentage of desired weight loss (independent variables) (DWL).

		Q1 § (*n* = 1492)	Q2 § (*n* = 1804)	Q3 § (*n* = 1470)	Q4 § (*n* = 1589)	
**MedDiet 17-items**		OR (95% CI)	OR (95% CI)	OR (95% CI)	OR (95% CI)	*P*
1: EVOO for cooking	*Crude OR*	1.00 (ref.)	1.23(1.04–1.45)	1.06(0.90–1.27)	1.14(0.96–1.35)	0.100
	*OR adjusted 1*	1.00 (ref.)	1.26(1.06–1.50)	1.13(0.93–1.36)	1.30(1.05–1.61)	0.030
	*OR adjusted 2*	1.00 (ref.)	1.24(1.04–1.48)	1.11(0.91–1.34)	1.28(1.04–1.59)	0.045
2: Vegetables	*Crude OR*	1.00 (ref.)	0.78(0.68–0.90)	0.81(0.70–0.94)	0.76(0.66–0.88)	0.001
	*OR adjusted 1*	1.00 (ref.)	0.78(0.68–0.91)	0.84(0.71–0.99)	0.80(0.67–0.96)	0.012
	*OR adjusted 2*	1.00 (ref.)	0.77(0.67–0.90)	0.84(0.71–0.99)	0.80(0.66–0.96)	0.008
3: Fruits	*Crude OR*	1.00 (ref.)	0.82(0.72–0.94)	0.82(0.71–0.95)	0.76(0.66–0.87)	0.001
	*OR adjusted 1*	1.00 (ref.)	0.86(0.74–0.99)	0.86(0.73–1.01)	0.80(0.67–0.96)	0.077
	*OR adjusted 2*	1.00 (ref.)	0.87(0.75–1.00)	0.87(0.74–1.03)	0.81(0.68–0.97)	0.133
4: Red and processed meat	*Crude OR*	1.00 (ref.)	0.84(0.73–0.97)	0.75(0.65–0.86)	0.82(0.71–0.95)	0.001
	*OR adjusted 1*	1.00 (ref.)	0.85(0.74–0.98)	0.78(0.66–0.92)	0.85(0.71–1.02)	0.022
	*OR adjusted 2*	1.00 (ref.)	0.85(0.73–0.98)	0.78(0.66–0.92)	0.85(0.71–1.02)	0.024
5: Butter, margarine, cream	*Crude OR*	1.00 (ref.)	0.99(0.83–1.18)	0.88(0.73–1.05)	0.85(0.71–1.01)	0.149
	*OR adjusted 1*	1.00 (ref.)	1.02(0.85–1.22)	0.91(0.75–1.11)	0.90(0.72–1.12)	0.535
	*OR adjusted 2*	1.00 (ref.)	1.02(0.85–1.23)	0.92(0.75–1.12)	0.91(0.73–1.13)	0.559
6: Sugar sweetened beverages	*Crude OR*	1.00 (ref.)	0.88(0.75–1.03)	0.81(0.69–0.96)	0.79(0.67–0.94)	0.029
	*OR adjusted 1*	1.00 (ref.)	0.89(0.75–1.05)	0.82(0.68–0.98)	0.81(0.66–0.99)	0.149
	*OR adjusted 2*	1.00 (ref.)	0.89(0.75–1.05)	0.82(0.68–0.98)	0.81(0.66–0.99)	0.147
7: Legumes	*Crude OR*	1.00 (ref.)	0.94(0.79–1.12)	0.87(0.73–1.05)	0.96(0.80–1.15)	0.533
	*OR adjusted 1*	1.00 (ref.)	0.97(0.81–1.16)	0.94(0.77–1.15)	1.06(0.85–1.33)	0.637
	*OR adjusted 2*	1.00 (ref.)	0.98(0.81–1.17)	0.95(0.77–1.16)	1.06(0.85–1.33)	0.678
8: Fish and seafood	*Crude OR*	1.00 (ref.)	1.09(0.95–1.26)	1.01(0.88–1.17)	0.95(0.82–1.09)	0.215
	*OR adjusted 1*	1.00 (ref.)	1.13(0.98–1.30)	1.05(0.89–1.23)	1.00(0.84–1.20)	0.284
	*OR adjusted 2*	1.00 (ref.)	1.13(0.98–1.31)	1.05(0.90–1.24)	1.01(0.84–1.20)	0.272
9: Sweets and pastries	*Crude OR*	1.00 (ref.)	0.96(0.83–1.10)	0.94(0.81–1.08)	0.85(0.74–0.98)	0.151
	*OR adjusted 1*	1.00 (ref.)	0.98(0.85–1.14)	1.00(0.84–1.18)	0.90(0.75–1.08)	0.598
	*OR adjusted 2*	1.00 (ref.)	0.98(0.85–1.14)	1.00(0.85–1.18)	0.90(0.75–1.08)	0.596
10: Nuts	*Crude OR*	1.00 (ref.)	1.07(0.93–1.22)	0.77(0.67–0.90)	0.68(0.58–0.78)	<0.001
	*OR adjusted 1*	1.00 (ref.)	1.14(0.98–1.32)	0.88(0.75–1.03)	0.85(0.71–1.02)	0.001
	*OR adjusted 2*	1.00 (ref.)	1.14(0.98–1.31)	0.88(0.75–1.03)	0.85(0.71–1.02)	0.001
11: Preference for white over red meat	*Crude OR*	1.00 (ref.)	0.94(0.80–1.10)	0.98(0.83–1.16)	0.97(0.82–1.14)	0.879
	*OR adjusted 1*	1.00 (ref.)	0.95(0.81–1.12)	1.03(0.85–1.23)	1.01(0.82–1.24)	0.802
	*OR adjusted 2*	1.00 (ref.)	0.96(0.81–1.13)	1.04(0.87–1.25)	1.02(0.83–1.25)	0.785
12: Sofrito	*Crude OR*	1.00 (ref.)	1.01(0.87–1.15)	0.96(0.83–1.11)	0.94(0.81–1.08)	0.701
	*OR adjusted 1*	1.00 (ref.)	1.03(0.89–1.19)	1.02(0.87–1.20)	1.06(0.88–1.26)	0.940
	*OR adjusted 2*	1.00 (ref.)	1.04(0.90–1.20)	1.03(0.88–1.21)	1.06(0.89–1.26)	0.934
13: Adding sugar to beverages	*Crude OR*	1.00 (ref.)	0.93(0.80–1.07)	1.06(0.91–1.23)	1.25(1.07–1.45)	0.001
	*OR adjusted 1*	1.00 (ref.)	0.85(0.73–0.99)	0.93(0.79–1.10)	1.00(0.83–1.20)	0.093
	*OR adjusted 2*	1.00 (ref.)	0.86(0.74–0.99)	0.95(0.80–1.12)	1.01(0.84–1.22)	0.112
14: White bread	*Crude OR*	1.00 (ref.)	0.88(0.77–1.01)	1.00(0.87–1.16)	0.98(0.85–1.13)	0.198
	*OR adjusted 1*	1.00 (ref.)	0.84(0.72–0.97)	0.97(0.82–1.14)	0.85(0.71–1.02)	0.048
	*OR adjusted 2*	1.00 (ref.)	0.83(0.72–0.97)	0.97(0.82–1.14)	0.85(0.70–1.02)	0.037
15: Whole grains	*Crude OR*	1.00 (ref.)	0.93(0.80–1.09)	1.01(0.86–1.19)	0.90(0.77–1.05)	0.398
	*OR adjusted 1*	1.00 (ref.)	0.91(0.78–1.07)	0.99(0.83–1.19)	0.85(0.70–1.04)	0.246
	*OR adjusted 2*	1.00 (ref.)	0.91(0.77–1.07)	1.00(0.83–1.19)	0.85(0.70–1.04)	0.215
16: Refined cereals	*Crude OR*	1.00 (ref.)	0.87(0.75–1.01)	0.91(0.78–1.06)	0.88(0.75–1.02)	0.251
	*OR adjusted 1*	1.00 (ref.)	0.85(0.73–0.99)	0.88(0.74–1.04)	0.79(0.65–0.96)	0.089
	*OR adjusted 2*	1.00 (ref.)	0.84(0.72–0.98)	0.87(0.73–1.04)	0.78(0.65–0.95)	0.066
17: Wine	*Crude OR*	1.00 (ref.)	1.16(0.99–1.36)	0.97(0.82–1.15)	0.84(0.71–1.00)	0.001
	*OR adjusted 1*	1.00 (ref.)	1.27(1.07–1.51)	1.11(0.91–1.35)	1.11(0.89–1.38)	0.045
	*OR adjusted 2*	1.00 (ref.)	1.25(1.05–1.49)	1.08(0.89–1.32)	1.10(0.88–1.37)	0.069

Abbreviations: OR. Odds Ratio. *OR adjusted 1*: Odds Ratio adjusted by sociodemographic characteristics (Age, gender, BMI, physical activity, diet, education level, marital status and smoking habit). *OR adjusted 2*: Odds Ratio adjusted by sociodemographic characteristics (Age, gender, BMI, physical activity, diet, education level, marital status and smoking habit) and presence of metabolic syndrome components. § Desired body weight loss = [(current body weight − ideal body weight)/current body weight] × 100. Due to the closeness of the cutting-percentiles, cut-offs were made considering a 5% of increase in desired body weight: Q1: <10% desired body weight loss; Q2: 10–15% desired body weight loss; Q3: 15–20% desired body weight loss; Q4: ≥20% desired body weight loss.

## Data Availability

There are restrictions on the availability of data for the PREDIMED-Plus trial, due to the signed consent agreements around data sharing, which only allow access to external researchers for studies following the project purposes. Requestors wishing to access the PREDIMED-Plus trial data used in this study can make a request to the PREDIMED-Plus trial Steering Committee chair: jordi.salas@urv.cat. The request will then be passed to members of the PREDIMED-Plus Steering Committee for deliberation.

## Supplementary Materials

The following are available online at https://www.mdpi.com/2072-6643/12/7/2114/s1, Table S1: Sociodemographic characteristics according to percentage of desired weight loss (DWL) in MEN, Table S2: Sociodemographic characteristics according to percentage of desired weight loss (DWL) in WOMEN, Table S3: Adherence to the Mediterranean Diet 17-items according to the percentage of desired weight loss (DWL) in MEN, Table S4. Adherence to the Mediterranean Diet 17-items according to the percentage of desired weight loss (DWL) in WOMEN.
