# Supplementary material for: Adherence to the Mediterranean Lifestyle and Desired Body Weight Loss in a Mediterranean Adult Population with Overweight: A PREDIMED-Plus Study
Source: Nutrients. 2020 Jul 16;12(7):2114. doi: 10.3390/nu12072114 (PMC7400596; doi:10.3390/nu12072114)
Supplement: Supplementary file 1 [file nutrients-12-02114-s001.pdf]

nutrients

MDPI

Adherence to the Mediterranean lifestyle and desired body weight loss in a Mediterranean adult population with overweight: PREDIMED-Plus Study

Cristina Bouzas[1,2,3], Maria del Mar Bibiloni[1,2,3], Alicia Julibert[1,2,3], Miguel Ruiz-Canela[1,4,], Jordi Salas-Salvadó[1,5], Dolores Corella[1,6], Maria Dolors Zomeño[1,7,8], Dora Romaguera[1,3], Jesús Vioque[9,10], Ángel M. Alonso-Gómez[1,11], Julia Wärnberg[1,12], J. Alfredo Martínez[1,13,14], Luís Serra-Majem[1,15], Ramon Estruch[1,16], Francisco J. Tinahones[1,17], José Lapetra[1,18], Xavier Pintó[1,19], Antonio García Ríos[1,20], Aurora Bueno-Cavanillas[10,21], José J. Gaforio[10,22], Pilar Matía-Martín[23], Lidia Daimiel[24], Vicente Martín-Sánchez[25,26], Josep Vidal[27], Clotilde Vázquez[1,28], Emilio Ros[1,29], Cesar Ignacio Fernandez-Lázaro[1,4], Nerea Becerra-Tomás[1,5], Ignacio Manuel Gimenez-Alba[1,6], Julia Muñoz[1,7], Marga Morey[1,3], Alejandro Oncina-Canovas[9,10], Lucas Tojal-Sierra[2,11], Jéssica Pérez-López[1,12], Itziar Abete[1,14], Tamara Casañas-Quintana[1,15], Sara Castro-Barquero[1,16], Rosa Bernal[1,17], José Manuel Santos-Lozano[1,18], Ana Galera[1,19], Escarlata Angullo-Martinez[1,2,3,30], F. Javier Basterra-Gortari[1,4,31], Josep Basora[1,5], Carmen Saiz[1,6], Olga Castañer[1,7], Marian Martín[1,3], Leyre Notario-Barandiarán[9,10], María C. Belló-Mora[1,11], Carmen Sayón-Orea[1,4,31], Jesús García-Gavilán[1,5], Albert Goday[1,7] and Josep A. Tur[1,2,3,*] on behalf of the PREDIMED-Plus investigators.

[1] CIBER Fisiopatología de la Obesidad y Nutrición (CIBEROBN), Instituto de Salud Carlos III (ISCIII), 28029 Madrid, Spain
[2] Research Group on Community Nutrition & Oxidative Stress, University of Balearic Islands, 07122 Palma de Mallorca, Spain
[3] Health Research Institute of the Balearic Islands (IdISBa), 07120 Palma de Mallorca, Spain
[4] Department of Preventive Medicine and Public Health, IdISNA, University of Navarra, 31008 Pamplona, Spain
[5] Human Nutrition Unit, Biochemistry and Biotechnology Department, IISPV, Hospital Universitari de Sant Joan, Universitat Rovira i Virgili, 43201 Reus, Spain
[6] Department of Preventive Medicine, University of Valencia, 46100 Valencia, Spain
[7] Unit of Cardiovascular Risk and Nutrition, Institut Hospital del Mar de Investigaciones Médicas Municipal d'Investigació Mèdica (IMIM), 08003 Barcelona, Spain
[8] Blanquerna School of Health Sciences. Universitat Ramon Llull, 08022 Barcelona, Spain
[9] Miguel Hernández University, ISABIAL-UMH, 46020 Alicante, Spain
[10] CIBER Epidemiología y Salud Pública (CIBERESP), Instituto de Salud Carlos III (ISCIII), 28029 Madrid, Spain
[11] Bioaraba Health Research Institute; Osakidetza Basque Health Service, Araba University Hospital, University of the Basque Country UPV/EHU, 48013 Vitoria-Gasteiz, Spain
[12] Department of Nursing, School of Health Sciences, University of Málaga-IBIMA, 29071 Málaga, Spain
[13] Precision Nutrition Program, IMDEA Food, CEI UAM + CSIC, 28049 Madrid, Spain
[14] Department of Nutrition, Food Sciences, and Physiology, Center for Nutrition Research, University of Navarra, 31008 Pamplona, Spain
[15] Institute for Biomedical Research, University of Las Palmas de Gran Canaria, 35016 Las Palmas de Gran Canaria, Spain
[16] Department of Internal Medicine, IDIBAPS, Hospital Clinic, University of Barcelona, 08036 Barcelona, Spain
[17] Virgen de la Victoria Hospital, Department of Endocrinology, Biomedical Research Institute of Málaga (IBIMA), University of Málaga, 29010 Málaga, Spain
[18] Department of Family Medicine, Research Unit, Distrito Sanitario Atención Primaria Sevilla, 41013 Sevilla, Spain
[19] Lipids and Vascular Risk Unit, Internal Medicine, Hospital Universitario de Bellvitge, Hospitalet de Llobregat, 08907 Barcelona, Spain

[20] Lipids and Atherosclerosis Unit, Department of Internal Medicine, Maimonides Biomedical Research Institute of Cordoba (IMIBIC), Reina Sofia University Hospital, University of Cordoba, 14004 Cordoba, Spain
[21] Department of Preventive Medicine, University of Granada, 18071 Granada, Spain
[22] Department of Health Sciences, Centro de Estudios Avanzados en Olivar y Aceites de Oliva, University of Jaen, 23071 Jaen, Spain
[23] Department of Endocrinology and Nutrition, Instituto de Investigación Sanitaria Hospital Clínico San Carlos (IdISSC), 28040 Madrid, Spain
[24] Nutritional Genomics and Epigenomics Group, IMDEA Food, CEI UAM + CSIC, 28049 Madrid, Spain
[25] CIBER Diabetes y Enfermedades Metabólicas (CIBERDEM), Instituto de Salud Carlos III (ISCIII), 28029 Spain
[26] Institute of Biomedicine (IBIOMED), University of León, 24071 León, Spain
[27] Department of Endocrinology, IDIBAPS, Hospital Clinic, University of Barcelona, 08036 Barcelona, Spain
[28] Department of Endocrinology, Fundación Jiménez-Díaz, 28040 Madrid, Spain
[29] Lipid Clinic, Department of Endocrinology and Nutrition, Institut d'Investigacions Biomèdiques August Pi Sunyer (IDIBAPS), Hospital Clínic, 08036 Barcelona, Spain
[30] Escola Graduada Primary Health Care Center, IBSalut, Palma de Mallorca, 07002Spain
[31] Servicio Navarro de Salud, Osasunbidea. 31071Pamplona, Spain
* Correspondence: **corresponding author:** Dr. Josep A. Tur (🖂), Research Group on Community Nutrition and Oxidative Stress,University of the Balearic Islands, IDISBA & CIBEROBN, Guillem Colom Bldg, Campus, E-07122 Palma de Mallorca, Spain.Phone: 34-971-1731; Fax: 34-971-173184; e-mail: pep.tur@uib.es

Abstract: Background. Body weight dissatisfaction is a hindrance to follow a healthy lifestyle and it has been associated to weight concerns. **Objectives.** The aim of this study was to assess the association between the adherence to Mediterranean lifestyle (diet and exercise) and the desired body weight loss in an adult Mediterranean population with overweight. **Methods.** Cross-sectional analysis in 6355 participants (3268 men; 3087 women) with metabolic syndrome and BMI (Body mass index) between 27.0-40.0 kg/m^2 (55-75 years old) from the PREDIMED-Plus trial. Desired weight loss was the percentage of weight that participants wish to lose. It was categorized into four cut-offs of this percentage (Q1: <10%, n=1495; Q2: 10-15%, n=1804; Q3: <15-20%, n=1470; Q4: ⩾20%, n=1589). Diet was assessed using a validated food frequency questionnaire and a 17-item Mediterranean diet questionnaire. Physical activity was assessed by the validated Minnesota-REGICOR and the validated Spanish version of the Nurses' Health Study questionnaire. **Results.** Participants reporting higher percentages of desired weight loss (Q3 and Q4) were younger, had higher real and perceived BMI and were more likely to have abdominal obesity. Desired weight loss correlated inversely to physical activity (Q1: 2106 MET min/week; Q4: 1585 MET min/week. p<0.001) and adherence to Mediterranean diet (Q1: 8.7; Q4: 8.3. p<0.001). **Conclusions.** In older Mediterranean individuals with weight excess, desired weight loss was inversely associated to Mediterranean lifestyle adherence. Deeply rooted aspects of MedDiet remained similar across groups. Longitudinal research is advised to be able to establish causality.

Keywords :Body image; Mediterranean lifestyle; overweight; obesity; older adults; desired weight loss; ideal weight; PREDIMED-Plus.

Annex 1. Supplemental Table 1. Sociodemographic characteristics according to percentage of desired weight loss (DWL) in MEN.

	Q1 § (n =830)		Q2 § (n =967)		Q3 § (n =761)		Q4 § (n =710)		
	Mean (SD)	Median (IQR)	Mean (SD)	Median (IQR)	Mean (SD)	Median (IQR)	Mean (SD)	Median (IQR)	P
Age (years)	64.7 (5.3)	65.0 (9.0) [a,b,c]	63.8 (5.1)	64.0 (9.0) [a,e]	63.5 (5.4)	63.0 (9.0) [b]	62.9 (5.3)	62.0 (8.0) [c,e]	<0.001
BMI (kg/m^2)	29.7 (2.0)	29.3 (2.6) [a,b,c]	31.4 (2.5)	31.1 (3.2) [a,d,e]	33.0 (2.7)	32.7 (3.7) [b,d,f]	35.3 (2.7)	35.3 (4.0) [c,e,f]	<0.001
Perceived BMI (kg/m^2)	29.8 (2.4)	29.5 (2.8) [a,b,c]	31.4 (2.6)	31.1 (3.5) [a,d,e]	33.0 (3.3)	32.6 (3.7) [b,d,f]	35.4 (3.2)	35.3 (4.1) [c,e,f]	<0.001
**Physical activity (PA)†**									
Total (PA) †	3178.9 (2743.8)	2517.5 (2907.7) [b,c]	2936.4 (2446.0)	2349.7 (3090.9) [e]	2748.5 (2595.2)	2097.9 (2580.4) [b]	2557.5 (2599.5)	1755.7 (2736.0) [c,e]	<0.001
Light PA †	823.5 (1010.7)	512.8 (1258.7)	750.8 (944.8)	419.6 (1118.9)	766.3 (997.1)	447.6 (1118.9)	832.3 (1073.4)	447.6 (1237.8)	0.215
Moderate PA †	1366.4 (1892.1)	839.2 (2030.9) [c]	1293.4 (1814.4)	559.4 (2097.9) [d,e]	1191.3 (1868.0)	524.5 (1678.3) [d,f]	980.9 (1681.5)	209.8 (1398.6) [c,e,f]	<0.001
Intense PA †	989.1 (1830.7)	111.9 (1272.7) [c]	892.2 (1478.8)	115.6 (1258.7) [e]	790.9 (1491.3)	111.9 (960.4)	744.3 (1480.6)	55.9 (702.1) [c,e]	<0.001
**Diet**									
Energy intake (kcal/d)	2494.9 (547.5)	2457.6 (767.0)	2518.3 (550.1)	2480.7 (776.5)	2520.8 (552.9)	2514.0 (777.2)	2529.9 (587.1)	2484.3 (777.8)	0.667
MedDiet Q score	8.4 (2.6) [c]	8.0 (4.0)	8.2 (2.7)	8.0 (4.0)	8.1 (2.7)	8.0 (4.0)	7.8 (2.6) [c]	8.0 (4.0)	<0.001
**BMI classification**	n (%)		n (%)		n (%)		n (%)		<0.001
Overweight (BMI < 30)	511 (54.8)		308 (33.0)		95 (10.2)		19 (2.0)		
Obesity (BMI > 30)	319 (13.8)		659 (28.5)		660 (28.5)		679 (29.3)		
**Education level**									0.178
Primary	319 (38.6)		366 (37.9)		280 (37.1)		294 (41.6)		
Secondary	263 (31.8)		306 (31.7)		271 (35.9)		230 (32.5)		
Tertiary	245 (29.6)		293 (30.4)		203 (26.9)		183 (25.9)		
**Marital status**									0.659
Married	718 (87.1)		820 (85.1)		652 (85.9)		593 (83.8)		
Divorced/separated	51 (6.2)		73 (7.6)		52 (6.9)		51 (7.2)		
Widower	28 (3.4)		29 (3.0)		21 (2.8)		27 (3.8)		
Other ‖	27 (3.3)		42 (4.4)		34 (4.5)		37 (5.2)		
Living alone ‡	47 (5.7)		77 (8.0)		46 (6.0)		53 (7.5)		0.178
**Smoking habit**									0.293
Current smoker	126 (15.3)		176 (18.2)		125 (16.6)		117 (16.5)		

Former smoker	496 (60.2)	582 (60.2)	463 (61.4)	450 (63.6)	
Never smoked	202 (24.5)	208 (21.5)	166 (22.0)	140 (19.8)	
**MetS components**					
High blood pressure	763 (91.9)	894 (92.5)	703 (92.4)	669 (94.2)	0.338
Hyperglycemia	633 (76.3)	748 (77.4)	589 (77.4)	560 (78.9)	0.684
Hypertriglyceridemia	493 (59.4)	566 (58.5)	459 (60.3)	423 (59.6)	0.902
Low HDL-cholesterol	354 (42.7)	375 (38.8)	302 (39.7)	284 (40.0)	0.395
Abdominal obesity	691 (83.3)	902 (93.3)	740 (97.2)	707 (99.6)	<0.001

Abbreviations: BMI. Body Mass Index. PA. Physical activity. MedDiet Q. 17-item Mediterranean Diet Questionnaire HDL-cholesterol. High density lipoprotein cholesterol. §Desired body weight loss= [(current body weight – ideal body weight) / current body weight] * 100. Due to the closeness of the cutting-percentiles, cut-offs were made considering a 5% of increase in desired body weight: Q1: <10% desired body weight loss; Q2: 10-15% desired body weight loss; Q3: 15-20% desired body weight loss; Q4: ≥20% desired body weight loss. †Measured in MET (Metabolic equivalent of task) min/week.‖ Other marital status were single and religious. ‡Living alone regardless of marital status.*Difference in means between groups were tested by one-way ANOVA and Bonferroni's post-hoc when normally distributed. or Kruskal-Wallis test when otherwise. Differences in prevalence's across groups were examined using χ^2. Different letters indicate statistically significant differences between groups (a, b, c, d, e, f) according to Bonferroni's post-hoc analysis.

Annex 1. Supplemental Table 2. Sociodemographic characteristics according to percentage of desired weight loss (DWL) in WOMEN.

	Q1 § (n =662)		Q2 § (n =837)		Q3 § (n =709)		Q4 § (n =879)		
	Mean (SD)	Median (IQR)	Mean (SD)	Median (IQR)	Mean (SD)	Median (IQR)	Mean (SD)	Median (IQR)	P
Age (years)	67.4 (4.2)	68.0 (7.0) [a,b,c]	66.5 (4.0)	66.0 (7.0) [a,e]	66.2 (3.9)	66.0 (6.0) [b,f]	65.4 (3.9)	65.0 (6.0) [c,e,f]	<0.001
BMI (kg/m^2)	30.1 (2.5)	29.7 (3.3) [a,b,c]	31.7 (3.0)	31.3 (4.3) [a,d,e]	33.4 (3.1)	33.3 (4.4) [b,d,f]	35.6 (3.0)	35.6 (4.7) [c,e,f]	<0.001
Perceived BMI (kg/m^2)	30.2 (2.9)	29.9 (3.6) [a,b,c]	31.7 (3.1)	31.3 (4.4) [a,d,e]	33.3 (3.4)	33.2 (4.6) [b,d,f]	35.5 (3.3)	35.4 (4.9) [c,e,f]	<0.001
**Physical activity (PA)†**									
Total (PA) †	2168.9 (1906.1)	1729.3 (2204.5) [c]	2176.1 (1904.1)	1717.5 (2237.8) [d,e]	2000.5 (1887.7)	1538.5 (2201.4) [d]	1863.1 (1743.3)	1421.9 (2042.0) [c]	<0.001
Light PA †	754.5 (926.8)	447.6 (1118.9)	757.5 (936.2)	447.6 (1118.9)	756.4 (896.4)	522.1 (1118.9)	699.5 (847.9)	447.6 (1044.3)	0.651
Moderate PA †	741.5 (1086.9)	209.8 (1165.5) [c]	723.5 (1077.9)	139.9 (1118.9) [e]	600.2 (978.2)	0.0 (909.1)	579.3 (1029.3)	0.0 (839.2) [c,e]	<0.001
Intense PA †	672.9 (1326.2)	83.9 (1049)	695.2 (1226.7)	83.9 (1118.9) [e]	644.0 (1257.2)	55.9 (839.2)	584.3 (1081.1)	55.9 (839.2) [e]	0.027
**Diet**									
Energy intake (kcal/d)	2193.4 (470.1)	2183.5 (675.4)	2194.3 (476.2)	2167.6 (655)	2221.1 (515.5)	2186.9 (705.2)	2216.3 (514.2)	2190.8 (736.3)	0.699
MedDiet Q score	9.1 (2.6) [b,c]	9.0 (4.0)	9.0 (2.6)	9.0 (4.0)	8.7 (2.6) [b]	9.0 (3.0)	8.7 (2.6) [c]	9.0 (4.0)	0.001
**BMI classification**	n (%)		n (%)		n (%)		n (%)		<0.001
Overweight (BMI < 30)	366 (47.3)		280 (36.2)		95 (12.3)		33 (4.3)		
Obesity (BMI > 30)	296 (13.0)		555 (24.3)		607 (26.6)		823 (36.1)		
**Education level**									<0.001
Primary	454 (69.1)		486 (58.7)		404 (57.9)		484 (56)		
Secondary	136 (20.7)		222 (26.8)		177 (25.4)		232 (26.8)		
Tertiary	67 (10.2)		120 (14.5)		117 (16.8)		149 (17.2)		
**Marital status**									0.002
Married	454 (68.8)		571 (68.4)		471 (66.6)		581 (66.2)		
Divorced/separated	38 (5.8)		70 (8.4)		64 (9.1)		89 (10.1)		
Widower	145 (22)		137 (16.4)		130 (18.4)		148 (16.9)		
Other ‖	23 (3.5)		57 (6.8)		42 (5.9)		59 (6.7)		
Living alone ‡	108 (16.3)		161 (19.2)		130 (18.3)		158 (18)		0.535
**Smoking habit**									<0.001
Current smoker	48 (7.3)		65 (7.8)		54 (7.6)		78 (8.9)		

Former smoker	121 (18.3)	188 (22.6)	198 (28.0)	262 (29.8)	
Never smoked	491 (74.4)	579 (69.6)	455 (64.4)	538 (61.3)	
**MetS components**					
High blood pressure	610 (92.1)	749 (89.5)	665 (93.8)	793 (90.2)	0.013
Hyperglycemia	505 (76.3)	582 (69.5)	507 (71.5)	671 (76.3)	0.002
Hypertriglyceridemia	342 (51.7)	434 (51.9)	378 (53.3)	446 (50.7)	0.789
Low HDL-cholesterol	303 (45.8)	377 (45)	302 (42.6)	410 (46.6)	0.429
Abdominal obesity	650 (98.2)	833 (99.5)	705 (99.4)	878 (99.9)	0.001

Abbreviations: BMI. Body Mass Index. PA. Physical activity. MedDiet Q. 17-item Mediterranean Diet Questionnaire HDL-cholesterol. High density lipoprotein cholesterol. §Desired body weight loss= [(current body weight – ideal body weight) / current body weight] * 100. Due to the closeness of the cutting-percentiles, cut-offs were made considering a 5% of increase in desired body weight: Q1: <10% desired body weight loss; Q2: 10-15% desired body weight loss; Q3: 15-20% desired body weight loss; Q4: ≥20% desired body weight loss. †Measured in MET (Metabolic equivalent of task) min/week.‖ Other marital status were single and religious. ‡Living alone regardless of marital status.*Difference in means between groups were tested by one-way ANOVA and Bonferroni's post-hoc when normally distributed. or Kruskal-Wallis test when otherwise. Differences in prevalence's across groups were examined using χ^2. Different letters indicate statistically significant differences between groups (a, b, c, d, e, f) according to Bonferroni's post-hoc analysis.

Annex 2. Supplemental Table 3. Adherence to the Mediterranean Diet 17-items according to the percentage of desired weight loss (DWL) in MEN.

	Q1 § (n =830)	Q2 § (n =967)	Q3 § (n =761)	Q4 § (n =710)	
**MedDiet 17-items**	n (%)	n (%)	n (%)	n (%)	*P*
1: EVOO for cooking	668 (80.5)	796 (82.3)	619 (81.3)	569 (80.1)	0.661
2: Vegetables	323 (38.9)	289 (29.9)	255 (33.5)	215 (30.3)	<0.001
3: Fruits	366 (44.1)	381 (39.4)	304 (39.9)	272 (38.3)	0.092
4: Red and processed meat	368 (44.3)	395 (40.8)	295 (38.8)	275 (38.7)	0.076
5: Butter, margarine, cream.	684 (82.4)	786 (81.3)	617 (81.1)	568 (80.0)	0.688
6: Sugar sweetened beverages	618 (74.5)	712 (73.6)	533 (70.0)	509 (71.7)	0.190
7: Legumes	176 (21.2)	190 (19.6)	145 (19.1)	158 (22.3)	0.391
8: Fish and seafood	378 (45.5)	442 (45.7)	329 (43.2)	274 (38.6)	0.016
9: Sweets and pastries	501 (60.4)	579 (59.9)	455 (59.8)	400 (56.3)	0.367
10: Nuts	375 (45.2)	445 (46.0)	300 (39.4)	239 (33.7)	<0.001
11: Preference white over red meat	576 (69.4)	642 (66.4)	535 (70.3)	453 (63.8)	0.029
12: Sofrito	483 (58.2)	543 (56.2)	444 (58.3)	406 (57.2)	0.772
13: Adding sugar to beverages	476 (57.3)	548 (56.7)	443 (58.2)	438 (61.7)	0.193
14: White bread	320 (38.6)	351 (36.3)	291 (38.2)	267 (37.6)	0.764
15: Whole grains	191 (23.0)	207 (21.4)	174 (22.9)	139 (19.6)	0.338
16: Refined cereals	232 (28.0)	233 (24.1)	211 (27.7)	164 (23.1)	0.054
17: Wine	270 (32.5)	354 (36.6)	246 (32.3)	219 (30.8)	0.065
**MedDiet Adherence**					
Low adherence (0-7)	308 (37.1)	406 (42.0)	323 (42.4)	318 (44.8)	0.001
Moderate adherence (8-10)	333 (40.1)	373 (38.6)	283 (37.2)	292 (41.1)	
High adherence (11-17)	189 (22.8)	188 (19.4)	155 (20.4)	100 (14.1)	

§ Desired body weight loss= [(current body weight – ideal body weight) / current body weight] * 100. Due to the closeness of the cutting-percentiles, cut-offs were made considering a 5% of increase in desired body weight:: Q1: <10% desired body weight loss; Q2: 10-15% desired body weight loss; Q3: 15-20% desired body weight loss; Q4: ≥20% desired body weight loss. Differences in prevalence's across groups were examined using χ^2.

Annex 2. Supplemental Table 4. Adherence to the Mediterranean Diet 17-items according to the percentage of desired weight loss (DWL) in WOMEN.

	Q1 § (n =662)	Q2 § (n =837)	Q3 § (n =709)	Q4 § (n =879)	
**MedDiet 17-items**	n (%)	n (%)	n (%)	n (%)	*P*
1: EVOO for cooking	484 (73.1)	658 (78.6)	532 (75.0)	693 (78.8)	0.020
2: Vegetables	286 (43.2)	342 (40.9)	271 (38.2)	331 (37.7)	0.111
3: Fruits	392 (59.2)	448 (53.5)	371 (52.3)	426 (48.5)	<0.001
4: Red and processed meat	404 (61.0)	461 (55.1)	359 (50.6)	470 (53.5)	0.001
5: Butter, margarine, cream.	525 (79.3)	674 (80.5)	544 (76.7)	678 (77.1)	0.207
6: Sugar sweetened beverages	537 (81.1)	642 (76.7)	548 (77.3)	653 (74.3)	0.018
7: Legumes	121 (18.3)	151 (18.0)	117 (16.5)	148 (16.8)	0.756
8: Fish and seafood	312 (47.1)	433 (51.7)	355 (50.1)	440 (50.1)	0.366
9: Sweets and pastries	407 (61.5)	500 (59.7)	416 (58.7)	505 (57.5)	0.437
10: Nuts	275 (41.5)	369 (44.1)	250 (35.3)	306 (34.8)	<0.001
11: Preference white over red meat	538 (81.3)	683 (81.6)	557 (78.6)	724 (82.4)	0.258
12: Sofrito	372 (56.2)	493 (58.9)	382 (53.9)	479 (54.5)	0.175
13: Adding sugar to beverages	464 (70.1)	556 (66.4)	502 (70.8)	642 (73.0)	0.027
14: White bread	368 (55.6)	425 (50.8)	388 (54.7)	458 (52.1)	0.207
15: Whole grains	227 (34.3)	274 (32.7)	242 (34.1)	273 (31.1)	0.488
16: Refined cereals	254 (38.4)	301 (36.0)	238 (33.6)	309 (35.2)	0.308
17: Wine	72 (10.9)	109 (13.0)	83 (11.7)	98 (11.1)	0.551
**MedDiet Adherence**					
Low adherence (0-7)	179 (27.0)	249 (29.7)	227 (32.0)	282 (32.1)	0.162
Moderate adherence (8-10)	288 (43.5)	346 (41.3)	308 (43.4)	370 (42.1)	
High adherence (11-17)	195 (29.5)	242 (28.9)	174 (24.5)	227 (25.8)	

§ Desired body weight loss= [(current body weight – ideal body weight) / current body weight] * 100. Due to the closeness of the cutting-percentiles, cut-offs were made considering a 5% of increase in desired body weight:: Q1: <10% desired body weight loss; Q2: 10-15% desired body weight loss; Q3: 15-20% desired body weight loss; Q4: ≥20% desired body weight loss. Differences in prevalence's across groups were examined using χ^2.

Annex 3. Supplemental Table 5. Association between the adherence to the Mediterranean Diet 17-items (dependent variables) and the percentage of desired weight loss (independent variables) (DWL) in MEN.

		Q1 § (n =830)	Q2 § (n =967)	Q3 § (n =761)	Q4 § (n =710)	
**MedDiet 17-items**		OR (95% CI)	OR (95% CI)	OR (95% CI)	OR (95% CI)	*P*
1: EVOO for cooking	*Crude OR*	1.00 (ref.)	1.13(0.89-1.43)	1.06(0.82-1.36)	0.98(0.76-1.26)	0.661
	OR adjusted 1	1.00 (ref.)	1.14(0.89-1.45)	1.07(0.81-1.41)	0.99(0.72-1.36)	0.676
	OR adjusted 2	1.00 (ref.)	1.12(0.87-1.44)	1.05(0.80-1.39)	0.99(0.72-1.36)	0.728
2: Vegetables	*Crude OR*	1.00 (ref.)	0.67(0.55-0.81)	0.79(0.64-0.97)	0.68(0.55-0.84)	<0.001
	OR adjusted 1	1.00 (ref.)	0.65(0.53-0.80)	0.77(0.61-0.97)	0.64(0.49-0.84)	<0.001
	OR adjusted 2	1.00 (ref.)	0.64(0.52-0.79)	0.76(0.61-0.96)	0.64(0.49-0.84)	<0.001
3: Fruits	*Crude OR*	1.00 (ref.)	0.82(0.68-0.99)	0.84(0.69-1.03)	0.79(0.64-0.97)	0.092
	OR adjusted 1	1.00 (ref.)	0.86(0.71-1.05)	0.89(0.71-1.11)	0.87(0.67-1.13)	0.520
	OR adjusted 2	1.00 (ref.)	0.88(0.72-1.08)	0.92(0.73-1.15)	0.89(0.69-1.15)	0.658
4: Red and processed meat	*Crude OR*	1.00 (ref.)	0.87(0.72-1.05)	0.79(0.65-0.97)	0.79(0.65-0.97)	0.077
	OR adjusted 1	1.00 (ref.)	0.92(0.75-1.12)	0.91(0.72-1.13)	0.96(0.74-1.25)	0.768
	OR adjusted 2	1.00 (ref.)	0.92(0.76-1.13)	0.92(0.73-1.15)	0.97(0.75-1.26)	0.806
5: Butter, margarine, cream	*Crude OR*	1.00 (ref.)	0.93(0.73-1.18)	0.91(0.71-1.18)	0.85(0.66-1.10)	0.688
	OR adjusted 1	1.00 (ref.)	0.97(0.76-1.25)	0.97(0.73-1.29)	0.94(0.68-1.30)	0.988
	OR adjusted 2	1.00 (ref.)	0.97(0.76-1.25)	0.98(0.73-1.30)	0.95(0.68-1.31)	0.989
6: Sugar sweetened beverages	*Crude OR*	1.00 (ref.)	0.96(0.78-1.18)	0.80(0.64-0.99)	0.87(0.69-1.09)	0.191
	OR adjusted 1	1.00 (ref.)	0.98(0.78-1.22)	0.81(0.64-1.04)	0.89(0.67-1.18)	0.325
	OR adjusted 2	1.00 (ref.)	0.99(0.79-1.23)	0.83(0.65-1.06)	0.90(0.68-1.20)	0.374
7: Legumes	*Crude OR*	1.00 (ref.)	0.91(0.72-1.14)	0.87(0.68-1.12)	1.06(0.83-1.36)	0.391
	OR adjusted 1	1.00 (ref.)	0.97(0.76-1.23)	0.97(0.74-1.28)	1.23(0.90-1.68)	0.289
	OR adjusted 2	1.00 (ref.)	0.97(0.76-1.24)	0.97(0.74-1.28)	1.23(0.90-1.68)	0.305
8: Fish and seafood	*Crude OR*	1.00 (ref.)	1.01(0.84-1.21)	0.91(0.75-1.11)	0.75(0.61-0.92)	0.016
	OR adjusted 1	1.00 (ref.)	1.06(0.88-1.29)	0.97(0.78-1.21)	0.83(0.64-1.07)	0.190
	OR adjusted 2	1.00 (ref.)	1.06(0.87-1.29)	0.97(0.77-1.21)	0.83(0.64-1.07)	0.208
9: Sweets and pastries	*Crude OR*	1.00 (ref.)	0.98(0.81-1.18)	0.98(0.80-1.19)	0.85(0.69-1.04)	0.367
	OR adjusted 1	1.00 (ref.)	1.00(0.81-1.22)	1.04(0.83-1.30)	0.91(0.70-1.18)	0.720
	OR adjusted 2	1.00 (ref.)	1.00(0.81-1.22)	1.04(0.82-1.30)	0.91(0.70-1.18)	0.718
10: Nuts	*Crude OR*	1.00 (ref.)	1.03(0.86-1.25)	0.79(0.65-0.96)	0.62(0.50-0.76)	<0.001
	OR adjusted 1	1.00 (ref.)	1.11(0.91-1.35)	0.90(0.72-1.13)	0.77(0.60-1.00)	0.020
	OR adjusted 2	1.00 (ref.)	1.10(0.90-1.34)	0.89(0.71-1.12)	0.77(0.59-0.99)	0.022
11: Preference for white over red meat	*Crude OR*	1.00 (ref.)	0.87(0.71-1.06)	1.04(0.84-1.29)	0.78(0.63-0.96)	0.029
	OR adjusted 1	1.00 (ref.)	0.90(0.73-1.10)	1.11(0.87-1.41)	0.86(0.66-1.12)	0.102
	OR adjusted 2	1.00 (ref.)	0.91(0.74-1.12)	1.13(0.89-1.43)	0.87(0.67-1.14)	0.099
12: Sofrito	*Crude OR*	1.00 (ref.)	0.92(0.76-1.11)	1.01(0.82-1.23)	0.96(0.78-1.18)	0.772
	OR adjusted 1	1.00 (ref.)	0.95(0.78-1.16)	1.09(0.88-1.37)	1.12(0.86-1.44)	0.464
	OR adjusted 2	1.00 (ref.)	0.96(0.79-1.17)	1.10(0.88-1.38)	1.12(0.87-1.45)	0.469
13: Adding sugar to beverages	*Crude OR*	1.00 (ref.)	0.97(0.81-1.17)	1.04(0.85-1.26)	1.20(0.98-1.47)	0.194
	OR adjusted 1	1.00 (ref.)	0.92(0.75-1.11)	0.95(0.76-1.19)	1.02(0.79-1.32)	0.715
	OR adjusted 2	1.00 (ref.)	0.89(0.73-1.09)	0.94(0.74-1.18)	1.02(0.78-1.32)	0.575
14: White bread	*Crude OR*	1.00 (ref.)	0.91(0.75-1.10)	0.99(0.81-1.21)	0.96(0.78-1.18)	0.764
	OR adjusted 1	1.00 (ref.)	0.92(0.75-1.12)	1.01(0.80-1.27)	0.94(0.72-1.23)	0.744
	OR adjusted 2	1.00 (ref.)	0.92(0.75-1.13)	1.01(0.80-1.28)	0.95(0.72-1.24)	0.750
15: Whole grains	*Crude OR*	1.00 (ref.)	0.91(0.73-1.14)	0.99(0.78-1.25)	0.81(0.64-1.04)	0.339
	OR adjusted 1	1.00 (ref.)	0.94(0.75-1.19)	1.08(0.83-1.40)	0.91(0.67-1.25)	0.580
	OR adjusted 2	1.00 (ref.)	0.95(0.75-1.20)	1.10(0.85-1.44)	0.93(0.68-1.28)	0.545
16: Refined cereals	*Crude OR*	1.00 (ref.)	0.82(0.66-1.01)	0.99(0.79-1.23)	0.77(0.61-0.98)	0.054
	OR adjusted 1	1.00 (ref.)	0.82(0.66-1.02)	0.96(0.75-1.22)	0.68(0.51-0.91)	0.022

	OR adjusted 2	1.00 (ref.)	0.81(0.65-1.02)	0.96(0.75-1.22)	0.68(0.51-0.92)	0.021
17: Wine	*Crude OR*	1.00 (ref.)	1.20(0.99-1.46)	0.99(0.80-1.22)	0.93(0.75-1.15)	0.065
	OR adjusted 1	1.00 (ref.)	1.25(1.02-1.54)	1.06(0.84-1.34)	1.00(0.76-1.32)	0.090
	OR adjusted 2	1.00 (ref.)	1.22(0.99-1.50)	1.03(0.81-1.30)	0.98(0.75-1.29)	0.135

Abbreviations: OR. Odds Ratio. *OR adjusted 1*: Odds Ratio adjusted by sociodemographic characteristics (Age, gender, BMI, physical activity, diet, education level, marital status and smoking habit). *OR adjusted 2*: Odds Ratio adjusted by sociodemographic characteristics (Age, gender, BMI, physical activity, diet, education level, marital status and smoking habit) and presence of metabolic syndrome components. [§]Desired body weight loss= [(current body weight – ideal body weight) / current body weight] * 100. Due to the closeness of the cutting-percentiles, cut-offs were made considering a 5% of increase in desired body weight:: Q1: <10% desired body weight loss; Q2: 10-15% desired body weight loss; Q3: 15-20% desired body weight loss; Q4: ≥20% desired body weight loss.

Annex 3. Supplemental Table 6. Association between the adherence to the Mediterranean Diet 17-items (dependent variables) and the percentage of desired weight loss (independent variables) (DWL) in WOMEN.

		Q1 § (n =662)	Q2 § (n =837)	Q3 § (n =709)	Q4 § (n =879)	
**MedDiet 17-items**		OR (95% CI)	OR (95% CI)	OR (95% CI)	OR (95% CI)	*P*
1: EVOO for cooking	*Crude OR*	1.00 (ref.)	1.35(1.07-1.72)	1.11(0.87-1.41)	1.37(1.08-1.74)	0.020
	OR adjusted 1	1.00 (ref.)	1.40(1.09-1.79)	1.18(0.91-1.54)	1.64(1.22-2.20)	0.003
	OR adjusted 2	1.00 (ref.)	1.36(1.06-1.75)	1.15(0.88-1.50)	1.62(1.21-2.18)	0.004
2: Vegetables	*Crude OR*	1.00 (ref.)	0.91(0.74-1.12)	0.81(0.66-1.01)	0.79(0.65-0.98)	0.112
	OR adjusted 1	1.00 (ref.)	0.93(0.75-1.15)	0.89(0.70-1.13)	0.94(0.73-1.21)	0.797
	OR adjusted 2	1.00 (ref.)	0.93(0.75-1.16)	0.90(0.71-1.15)	0.93(0.72-1.20)	0.864
3: Fruits	*Crude OR*	1.00 (ref.)	0.79(0.65-0.97)	0.76(0.61-0.94)	0.65(0.53-0.79)	0.001
	OR adjusted 1	1.00 (ref.)	0.84(0.68-1.04)	0.81(0.64-1.02)	0.74(0.57-0.95)	0.124
	OR adjusted 2	1.00 (ref.)	0.83(0.67-1.04)	0.81(0.64-1.03)	0.73(0.57-0.94)	0.111
4: Red and processed meat	*Crude OR*	1.00 (ref.)	0.78(0.64-0.96)	0.66(0.53-0.81)	0.73(0.60-0.90)	0.001
	OR adjusted 1	1.00 (ref.)	0.78(0.63-0.96)	0.66(0.52-0.83)	0.74(0.57-0.95)	0.007
	OR adjusted 2	1.00 (ref.)	0.77(0.62-0.95)	0.65(0.51-0.82)	0.73(0.57-0.95)	0.005
5: Butter, margarine, cream	*Crude OR*	1.00 (ref.)	1.08(0.84-1.39)	0.86(0.67-1.11)	0.88(0.69-1.12)	0.208
	OR adjusted 1	1.00 (ref.)	1.08(0.82-1.40)	0.85(0.64-1.13)	0.87(0.64-1.17)	0.264
	OR adjusted 2	1.00 (ref.)	1.09(0.83-1.42)	0.85(0.64-1.13)	0.88(0.64-1.19)	0.252
6: Sugar sweetened beverages	*Crude OR*	1.00 (ref.)	0.77(0.60-0.99)	0.79(0.61-1.03)	0.67(0.53-0.86)	0.018
	OR adjusted 1	1.00 (ref.)	0.77(0.59-0.99)	0.80(0.60-1.07)	0.71(0.52-0.96)	0.130
	OR adjusted 2	1.00 (ref.)	0.77(0.59-1.00)	0.80(0.60-1.07)	0.71(0.52-0.96)	0.133
7: Legumes	*Crude OR*	1.00 (ref.)	0.98(0.76-1.28)	0.88(0.67-1.17)	0.91(0.69-1.18)	0.757
	OR adjusted 1	1.00 (ref.)	1.00(0.76-1.32)	0.93(0.68-1.26)	0.97(0.70-1.34)	0.949
	OR adjusted 2	1.00 (ref.)	1.00(0.76-1.32)	0.92(0.68-1.26)	0.97(0.70-1.34)	0.943
8: Fish and seafood	*Crude OR*	1.00 (ref.)	1.20(0.98-1.47)	1.12(0.91-1.39)	1.12(0.92-1.38)	0.366
	OR adjusted 1	1.00 (ref.)	1.21(0.98-1.50)	1.15(0.91-1.46)	1.22(0.95-1.56)	0.311
	OR adjusted 2	1.00 (ref.)	1.23(0.99-1.52)	1.16(0.92-1.47)	1.22(0.95-1.57)	0.275
9: Sweets and pastries	*Crude OR*	1.00 (ref.)	0.93(0.75-1.15)	0.89(0.72-1.10)	0.85(0.69-1.04)	0.437
	OR adjusted 1	1.00 (ref.)	0.96(0.77-1.21)	0.97(0.76-1.23)	0.91(0.70-1.19)	0.920
	OR adjusted 2	1.00 (ref.)	0.95(0.76-1.19)	0.96(0.75-1.23)	0.90(0.70-1.18)	0.892
10: Nuts	*Crude OR*	1.00 (ref.)	1.11(0.90-1.36)	0.77(0.62-0.95)	0.75(0.61-0.93)	<0.001
	OR adjusted 1	1.00 (ref.)	1.15(0.93-1.43)	0.83(0.65-1.05)	0.89(0.69-1.16)	0.022
	OR adjusted 2	1.00 (ref.)	1.15(0.92-1.43)	0.83(0.65-1.05)	0.89(0.69-1.16)	0.022
11: Preference for white over red meat	*Crude OR*	1.00 (ref.)	1.02(0.79-1.33)	0.84(0.65-1.10)	1.08(0.83-1.40)	0.259
	OR adjusted 1	1.00 (ref.)	1.03(0.79-1.36)	0.91(0.68-1.21)	1.20(0.87-1.65)	0.227
	OR adjusted 2	1.00 (ref.)	1.02(0.78-1.34)	0.90(0.67-1.20)	1.20(0.87-1.65)	0.199
12: Sofrito	*Crude OR*	1.00 (ref.)	1.12(0.91-1.37)	0.91(0.74-1.13)	0.93(0.76-1.14)	0.175
	OR adjusted 1	1.00 (ref.)	1.15(0.93-1.43)	0.97(0.77-1.23)	1.03(0.80-1.33)	0.378
	OR adjusted 2	1.00 (ref.)	1.15(0.93-1.43)	0.97(0.77-1.23)	1.03(0.80-1.33)	0.376
13: Adding sugar to beverages	*Crude OR*	1.00 (ref.)	0.84(0.68-1.05)	1.03(0.82-1.31)	1.16(0.92-1.45)	0.027
	OR adjusted 1	1.00 (ref.)	0.76(0.61-0.96)	0.89(0.69-1.15)	0.95(0.72-1.25)	0.085
	OR adjusted 2	1.00 (ref.)	0.80(0.63-1.01)	0.93(0.72-1.21)	0.98(0.74-1.30)	0.179
14: White bread	*Crude OR*	1.00 (ref.)	0.82(0.67-1.01)	0.97(0.78-1.19)	0.87(0.71-1.06)	0.207
	OR adjusted 1	1.00 (ref.)	0.75(0.60-0.93)	0.90(0.71-1.15)	0.75(0.58-0.96)	0.026
	OR adjusted 2	1.00 (ref.)	0.73(0.59-0.91)	0.90(0.71-1.14)	0.73(0.57-0.95)	0.015
15: Whole grains	*Crude OR*	1.00 (ref.)	0.93(0.75-1.16)	0.99(0.79-1.24)	0.86(0.70-1.07)	0.489
	OR adjusted 1	1.00 (ref.)	0.88(0.71-1.11)	0.92(0.72-1.17)	0.79(0.61-1.03)	0.344
	OR adjusted 2	1.00 (ref.)	0.87(0.70-1.09)	0.91(0.71-1.17)	0.78(0.60-1.02)	0.305
16: Refined cereals	*Crude OR*	1.00 (ref.)	0.90(0.73-1.11)	0.81(0.65-1.01)	0.87(0.71-1.07)	0.309
	OR adjusted 1	1.00 (ref.)	0.85(0.68-1.06)	0.77(0.60-0.98)	0.83(0.64-1.07)	0.210

	OR adjusted 2	1.00 (ref.)	0.84(0.68-1.05)	0.77(0.60-0.98)	0.82(0.63-1.07)	0.209
17: Wine	*Crude OR*	1.00 (ref.)	1.23(0.89-1.68)	1.09(0.78-1.52)	1.03(0.74-1.42)	0.552
	OR adjusted 1	1.00 (ref.)	1.28(0.92-1.78)	1.19(0.82-1.71)	1.27(0.85-1.89)	0.512
	OR adjusted 2	1.00 (ref.)	1.29(0.93-1.80)	1.18(0.82-1.71)	1.28(0.86-1.91)	0.470

Abbreviations: OR. Odds Ratio. *OR adjusted 1*: Odds Ratio adjusted by sociodemographic characteristics (Age, gender, BMI, physical activity, diet, education level, marital status and smoking habit). *OR adjusted 2*: Odds Ratio adjusted by sociodemographic characteristics (Age, gender, BMI, physical activity, diet, education level, marital status and smoking habit) and presence of metabolic syndrome components. §Desired body weight loss= [(current body weight – ideal body weight) / current body weight] * 100. Due to the closeness of the cutting-percentiles, cut-offs were made considering a 5% of increase in desired body weight:: Q1: <10% desired body weight loss; Q2: 10-15% desired body weight loss; Q3: 15-20% desired body weight loss; Q4: ≥20% desired body weight loss.
